# Simultaneous expression of flotillin-1, flotillin-2, stomatin and caveolin-1 in non-small cell lung cancer and soft tissue sarcomas

**DOI:** 10.1186/1471-2407-14-100

**Published:** 2014-02-17

**Authors:** Ksenia A Arkhipova, Anastasia N Sheyderman, Konstantin K Laktionov, Valeria V Mochalnikova, Irina B Zborovskaya

**Affiliations:** 1 Laboratory for Cellular and Viral Oncogene Regulation, Carcinogenesis Research Institute, N. N. Blokhin Russian Cancer Research Center RAMS, 24, Kashirskoye sh., Moscow 115478, Russia; 2 Thoraco-Abdominal Oncology Department, Clinical Oncology Research Institute, N. N. Blokhin Russian Cancer Research Center RAMS, 24, Kashirskoye sh., Moscow 115478, Russia; 3 Human Tumor Pathologic Anatomy Department, Clinical Oncology Research Institute, N. N. Blokhin Russian Cancer Research Center RAMS, 24, Kashirskoye sh., Moscow 115478, Russia

**Keywords:** Flotillin, Stomatin, Caveolin, Non-small cell lung cancer, Soft tissue sarcoma

## Abstract

**Background:**

At the present time, there is a lack of data about the involvement of flotillins and stomatin in the development of non-small cell lung cancer (NSCLC) and soft tissue sarcomas (STS). Moreover, changes in expression of members of different families of the microdomain-forming proteins (caveolins and SPFH-domain containing family) are usually investigated independently of each other. In this study we performed a combined analysis of flotillins, stomatin, and caveolin-1 expression in these pathologies and evaluated correlations between generated data and clinicopathological characteristics of the specimens.

**Methods:**

The protein and mRNA expression was analyzed by Western blotting and real-time PCR, respectively, in tissue specimens of patients undergoing surgery for non-small cell lung cancer and soft tissue sarcomas. Association between expression of studied proteins and patient clinicopathological characteristics or outcome was evaluated.

**Results:**

Stomatin protein expression was down-regulated in 80% of NSCLC samples and this decrease significantly associated with presence of lymph node metastases. Flotillin-2 protein expression was up-regulated in the majority of NSCLC samples whereas caveolin-1α expression was decreased. We revealed a strong correlation between *STOM* and *FLOT-1* mRNA expression in both pathologies, although the gene expression changes were diverse.

**Conclusions:**

Our data demonstrate for the first time that expression of stomatin, a poorly studied microdomain-forming protein, significantly changes in human tumors, thus pointing to its importance in the progression of NSCLC. We also suggest the existence of some relationship between the expression of these proteins.

## Background

Recently, the studies of the lipid rafts - membrane microdomains enriched with sphingolipids and cholesterol, as well as a wide range of proteins, - have started to attract increasing interest. A special type of lipid rafts is microdomains stabilized by microdomain-forming proteins (MFP), such as caveolins and SPFH (**S**tomatins, **P**rohibitins, **F**lotillins, **H**flK/C) domain-containing proteins. The caveolin family is one of the best studied and the role of caveolin-1 is mainly determined by its ability to form signalosomes, i.e. not only to support the integrity of lipid rafts, but also, due to interaction with many residential signaling molecules, to coordinate and regulate signal transduction in the cell [1]. As a result caveolin-1 can affect cell proliferation, programmed cell death, migration and other processes important for tumor transformation and progression. To date, the analysis of caveolin-1 expression has been carried out in a wide range of tumors and cell lines of various origins. It was shown that, depending on the histogenesis of the tumor, caveolin-1 may function as a tumor suppressor gene as well as an oncogene.

The role of the SPFH superfamily in carcinogenesis has been studied less extensively. Proteins of this superfamily, such as flotillins and stomatin, share a number of common features with caveolins. They are also widely expressed in human tissues, primarily localized within the plasma membrane, have similar topology, capability for oligomerization and actively participate in the regulation of signaling pathways, some of which intersect with caveolin-dependent pathways. In the normal cell physiology flotillins are involved in neuronal regeneration, clathrin-independent endocytosis, glucose uptake, etc. [2-4]. Hazarika *et al.* demonstrated that metastasizing melanomas are characterized by increased flotillin-2 expression. Moreover, the exogenous flotillin-2 expression in melanoma cells leads to the acquisition of metastasizing phenotype [5]. It has also been demonstrated that flotillin-1 plays an important role in cellular proliferation, and its increased expression correlates with poor outcome in patients with breast cancer and lung adenocarcinomas [6-8]. Stomatin was first discovered as an essential component of erythrocyte cellular membranes, and its absence was related to the development of hereditary hemolytic anemia [9]. Stomatin is also expressed widely in the human tissues; however, its functions have been studied only scantily. It is known that stomatin modulates the activity of acid-sensing ion channels [10] and influences glucose uptake [11]. At the present time, there are no data on the role of stomatin in carcinogenesis and no information about stomatin expression in human tumors.

Lung cancer is the leading cause of cancer deaths worldwide among both men and women. Identification of the molecular markers determining the risk of occurrence and progression and approaches for therapeutic treatment of lung cancer are the most significant important problems in molecular oncology. On the contrary, soft tissue sarcomas (STS) have not been studied nearly as extensive as lung cancer. However, this group of tumors is quite diverse; there are over 100 histological variants with individual clinical, prognostic and therapeutic features, which make the study of this type of tumors extremely important.

Here we present novel data on mRNA and protein expression of stomatin, flotillin-1 and −2 in human adenocarcinoma and squamous cell lung carcinoma specimens. We also examined mRNA expression of MFP and caveolin-1α protein in the STS group. To our knowledge, this is the first study to simultaneously investigate the protein expression of members of different MFP families in human tumors of epithelial and mesenchymal origin. Our results suggest some relationship between these proteins and the existence of a strong correlation between *STOM* and *FLOT-1* mRNA expression, observed in both groups.

## Results

### Expression of microdomain-forming proteins in NSCLC

Here and later in this paper, by the term “down-regulation” we mean “down-regulation in tumor samples compared with corresponding normal tissue samples”, by “up-regulation” we mean “up-regulation in tumor samples compared with normal tissue samples” and by “equal expression” we mean “equal expression levels in tumor and normal tissue samples”.

We investigated the mRNA expression of flotillin-1, stomatin, and caveolin-1 using real-time PCR in 22 paired (tumor and corresponding normal tissue) samples of adenocarcinomas and 26 paired samples of squamous cell carcinomas (Additional file 1). The expression of all investigated microdomain-forming proteins was down-regulated in the majority of specimens. There were no significant differences in the expression of these genes in groups of samples divided according to clinicopathological characteristics (Table 1).

**Table 1:** **Expression**^**a **^**of microdomain-forming proteins mRNA in NSCLC**

	**Stomatin (n = 48)**			**Flotillin-1 (n = 47)**			**Caveolin-1 (n = 48)**	
	**up**	**equal**	**down**	**up**	**equal**	**down**	**equal**	**down**
NSCLC	3	8	37	3	11	33	2	46
Adenocarcinomas	2	4	16	2	6	13	1	21
Squamous cell carcinomas	1	4	21	1	5	20	1	25
Tumor size								
T1-T2	3	3	24	2	6	21	1	29
T3-T4	0	5	13	1	5	12	1	17
Lymph node status								
N0	1	3	16	0	7	12	0	20
N+	2	5	21	3	4	21	2	26
Clinical stage								
I-II	2	2	22	1	6	18	1	25
III-IV	1	6	15	2	5	15	1	21
Degree of differentiation								
High	1	0	1	0	0	1	0	2
Moderate	1	5	24	2	6	22	1	29
Low	1	3	12	1	5	10	1	15

^a^up – higher gene expression in tumors compared with normal tissue samples.

down – lower gene expression in tumors compared with normal tissue samples.

equal – no significant difference in gene expression in tumors versus normal tissue samples.

We performed a correlation analysis of caveolin-1, stomatin, and flotillin-1 mRNA expression in the whole group of non-small cell lung cancer (NSCLC) specimens and in its subgroups in accordance with the clinicopathological characteristics of the specimens (Table 2). We used Spearman's rank correlation coefficient to assess strength of relationships between expression changes of studied genes; the higher the absolute value of the correlation coefficient (it changes from −1 to 1), the stronger the linear relationship and the two variables tend to increase or decrease together. Expression of stomatin and flotillin-1 demonstrated the strongest correlation which varied insignificantly in different groups. The correlation between the expression levels of caveolin-1 and flotillin-1 was found in groups of patients with small tumors and early clinical stages where it was stronger than in the whole group of NSCLC specimens. The most attention drew the correlation between caveolin-1 and stomatin expression because it emerged in groups of patients with favorable characteristics such as small tumor size (r = 0,666, p < 0,01, Spearman’s rank correlation), absence of lymph nodal metastases (r = 0,575, p < 0,01), high and moderate differentiation degree (r = 0,463, p < 0,01) and early stage of disease (r = 0,672, p < 0,01). It also should be noted that the two main histological types of NSCLC (adenocarcinomas and squamous cell carcinomas) did not differ in correlations between the expression of the studied genes.

**Table 2:** Spearman’s rank correlations between caveolin-1, stomatin and flotillin-1 mRNA expression in groups of tumors, divided according to clinicopathological characteristics

**Group**	**Spearman's rank correlation coefficients**		
	**Caveolin-1 and Stomatin**	**Caveolin-1 and Flotillin-1**	**Stomatin and Flotillin-1**
Histology			
NSCLC	0,437	0,295^a^	0,849
Adenocarcinomas	0,469	NS	0,848
SCC	0,45	NS	0,816
Clinical stage			
I-II	0,672	0,442^b^	0,863
III-IV	NS^c^	NS	0,869
Tumor stage			
T1-T2	0,666	0,497	0,886
T3-T4	NS	NS	0,777
Lymph node status			
Positive	NS	NS	0,885
Negative	0,575	NS	0,901
Degree of differentiation			
Moderate	0,463	NS	0,864
Poor	NS	NS	0,815

NSCLC – non-small cell lung cancer.

SCC – squamous cell carcinoma.

^a^p = 0.044.

^b^p = 0.027, for others – p < 0.01.

NS – statistically non-significant.

**Figure 1:**
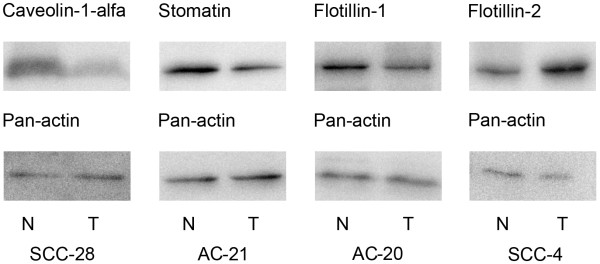
**Western blot analysis of expression of microdomain-forming proteins in paired samples of NSCLC.** Actin was used as a loading control. These representative samples illustrate the main trends of changes in transcription and protein expression. T – tumor tissue, N – normal tissue, SCC – squamous cell carcinoma, AC - adenocarcinoma.

To investigate stomatin, flotillin-1, flotillin-2, and caveolin-1α protein expression in NSCLC we performed Western blot analysis (Figure 1). Expression of all MFP was detected in all examined specimens, both in tumors and normal ones. The results of the analysis and correlations with clinical and pathological characteristics are represented in Table 3. Stomatin protein expression was decreased in 80% of tumor samples compared to corresponding normal tissue samples and its down-regulation was associated with positive lymph nodal status (p < 0,05, χ^2^-test). Protein expression of flotillin-2 was up-regulated in 53% of tumor samples compared to their normal tissue, and a high level of flotillin-2 was correlated with high and moderate differentiation degree (p < 0,05, χ^2^-test). Analysis of the flotillin-1 protein showed that its expression was decreased and increased in approximately equal amounts of specimens, in 38% and 40%, respectively. Moreover, these groups had similar clinicopathological characteristics and survival rates. Caveolin-1α protein expression was decreased in 75% of samples and in all others it was unchanged. We also found a correlation between the small tumors and the equal amounts of caveolin-1α in tumor and normal tissues (p < 0,05, Fisher’s exact test). Another important observation was that within the group of 12 paired specimens with equal expression of caveolin-1α, 11 were small size tumors (T1-T2) and, furthermore, 7 out of these 11 developed metastases in the lymph nodes.

**Table 3:** **Associations between expression**^**a **^**of microdomain-forming proteins and clinicopathological characteristics of NSCLC patients**

	**Stomatin**			**Flotillin-1**			**Flotillin-2**			**Caveolin-1**	
	**up**	**equal**	**down**	**up**	**equal**	**down**	**up**	**equal**	**down**	**equal**	**down**
Stomatin											
up	4										
equal		4									
down			33								
*p*											
Flotillin-1											
up	3	0	12	18							
equal	1	1	4		10						
down	0	2	14			17					
*p*	0,242										
Flotillin-2											
up	3	2	15	11	7	5	24				
equal	0	2	9	4	2	5		13			
down	0	0	6	1	1	5			8		
*p*	0,39			0,176							
Caveolin-1											
equal	1	1	6	5	3	1	4	6	1	12	
down	3	3	26	13	6	16	19	7	7		36
*p*	0,925			0,155			0,107				
Tumor size											
T1-T2	4	1	20	12	7	10	12	11	5	11	20
T3-T4	0	3	13	6	3	7	12	2	3	1	16
*p*	0,094			0,816			0,117			**0,036**^**b**^	
Lymph node status											
N0	3	4	12	7	3	9	9	8	1	5	15
N+	1	0	21	11	7	8	15	5	7	7	21
*p*	**0,026**^**c**^			0,476			0,078			1	
Clinical stage											
I-II	3	2	18	10	4	11	11	9	3	8	18
III-IV	1	2	15	8	6	6	13	4	5	4	18
*p*	0,714			0,459			0,277			0,505	
Degree of differentiation											
moderate	3	4	22	10	8	11	17	11	2	10	22
low	1	0	11	8	2	6	7	2	7	2	14
*p*	0,377			0,432			**0,007**^**c**^			0,289	

^a^up – higher gene expression in tumors compared with normal tissue samples.

down – lower gene expression in tumors compared with normal tissue samples.

equal – no significant difference in gene expression in tumors versus normal tissue samples.

^b^Fisher’s exact test.

^c^χ^2^ test.

Data in bold represents statistically significant values.

To assess the prognostic significance of MFP expression changes we carried out a log-rank analysis of the Kaplan-Meier survival curves for 35 patients. Although we analyzed all the possible groups of samples (taking into account expression changes of MFP and clinicopathological characteristics), statistically significant differences were detected only in groups of specimens divided by stage (I-II vs. III-IV, p < 0,05, log-rank test) and by tumor size (T1-2 vs. T3-4, p < 0,05), which is obvious. The Cox’ univariant regression analysis was used to assess the mortality hazard ratio which for patients with advance stage of disease (III-IV) was HR = 3.854 (95.0% CI 1.247-11.909, p < 0,05), and for patients with larger size of tumors (Т3-4) was HR = 5.007 (95.0% CI 1.848-13.564, p < 0,05).

### Expression of microdomain-forming proteins in soft tissue sarcomas

We studied mRNA expression of caveolin-1, stomatin, and flotillin-1 by real-time PCR in 37 paired samples, and protein expression of caveolin-1α in 35 paired samples from the STS group. The evaluation of mRNA expression was performed only in the group of malignant tumors and the results are represented in Table 4. As follows from the table, stomatin mRNA expression increased in the majority of the mesenchymal tumor specimens. However, such up-regulation is more typical for malignant fibrous histiocytoma, one of the most aggressive types of STS, where out of 7 studied specimens only in one case stomatin mRNA levels were equal in normal and tumor tissues. We also found differences in mRNA expression of caveolin-1 and flotillin-1 between liposarcomas and other mesenchymal tumors (p < 0,05, χ^2^-test, Table 5). Correlation analysis revealed strong relationships between mRNA expression of stomatin and flotillin-1 (r = 0,666, p < 0.01, Spearman’s rank correlation), caveolin-1 and flotillin-1 (r = 0,492, p < 0.01), and a weaker one between stomatin and caveolin-1 (r = 0.338, р = 0.047).

**Table 4:** **Expression**^**a **^**of microdomain-forming proteins mRNA in STS**

	**Gene expression in tumors compared with normal tissue samples**								
	**Stomatin**			**Flotillin-1**			**Caveolin-1**		
	**up**	**equal**	**down**	**up**	**equal**	**down**	**up**	**equal**	**down**
Liposarcomas	5	5	5	3	3	8	0	3	12
Synovial sarcoma	3	1	2	4	2	0	3	0	3
Malignant fibrous histiocytoma	6	1	0	5	0	2	4	1	2
Malignant schwannoma	2	0	2	3	0	1	1	2	1
Other malignant tumors	2	0	1	1	1	1	2	0	1

^a^up – higher gene expression in tumors compared with normal tissue samples.

down – lower gene expression in tumors compared with normal tissue samples.

equal – no significant difference in gene expression in tumors versus normal tissue samples.

**Table 5:** **Differences in mRNA expression**^**a **^**of microdomain-forming proteins between liposarcomas and other malignant soft tissue sarcomas**

	**Gene expression in tumors compared with normal tissue samples**								
	**Stomatin**			**Flotillin-1**			**Caveolin-1**		
	**up**	**equal**	**down**	**up**	**equal**	**down**	**up**	**equal**	**down**
Liposarcomas	5	5	5	3	3	8	0	3	12
Other malignant tumors	13	2	5	13	3	4	10	3	7
*p*	<0,01^b^			<0,05^b^			NS		

^a^up – higher gene expression in tumors compared with normal tissue samples.

down – lower gene expression in tumors compared with normal tissue samples.

equal – no significant difference in gene expression in tumors versus normal tissue samples.

^b^χ^2^ test.

NS – statistically non-significant.

We examined the caveolin-1α protein expression levels in benign and malignant tumors. The expression of caveolin-1α was decreased in samples from 23 of 29 (79,3%) patients with malignant tumors, in 3 (10,3%) cases caveolin-1α expression was increased, and there were no difference in expression levels of caveolin-1α between normal and tumor tissues in 3 (10,3%) other cases. Analysis of specimens from 5 patients with benign tumors showed no difference in protein expression of caveolin-1α between normal and tumor tissues.

## Discussion

### Expression changes of MFP correlate with clinicopathological characteristics of specimens

In this work, we demonstrated for the first time that stomatin mRNA and protein expression changes in tumor specimens of patients with NSCLC and soft tissue sarcomas. As there is a lack of data about stomatin participation in the main cancer-related signaling pathways, it was especially interesting to found out its association with positive lymph node metastasis status of patients with NSCLC. Our data indicate that decreased stomatin expression is an unfavorable factor for lung cancer; however, the mechanisms of its action are unclear. Two possible explanations for the down-regulation of stomatin are that it is due to transcriptional regulation or change in the methylation status of its promoter. These explanations are quite plausible, as we observed a significant decrease in both mRNA and protein expression levels of stomatin in the majority of tumor specimens.

Flotillin-2 protein up-regulation was detected in a half of the studied samples, while down-regulation was observed in 30%. Hazarika *et al.* showed that increased expression of flotillin-2 was also typical for melanomas, especially for the more aggressive metastasizing forms [5]. We did not find association between changes in expression of flotillin-2 and lymph node status, nevertheless, we demonstrated correlation between its changes and degree of differentiation. This fact may be explained by findings of previous *in vitro* studies, which described a direct relationship between degree of differentiation and the expression rates of flotillin-2. Volonte *et al.* showed that flotillin-2 up-regulates during differentiation of skeletal myoblast cell line C2C12 [12]. A similar result was obtained by Bickel *et al.* for 3 T3-L1 adipocyte cell line [13]. By analyzing flotillin-1 protein expression, we identified two equally sized groups (with increased and down-regulated expression), which had similar clinical and pathological parameters. Furthermore, our findings contradict those reported by Zhang *et al.*[8], who detected flotillin-1 up-regulation in the majority of studied samples of lung adenocarcinomas and demonstrated its correlation with lymph node metastases. As we did not find any differences in flotillin-1 expression between adenocarcinomas and squamous cell carcinomas, we believe that this contradiction is due to differences in sampling, methodology of investigation, or population specifics. We also detected differences between mRNA and protein expression of flotillin-1 in our samples, which may be explained by post-translation regulation or protein stability.

The results of caveolin-1α expression analysis agree quite well with previously reported data for both groups of tumors [14-18]. Immunohistochemical analysis of NSCLC detected caveolin-1 expression in 15-30% of specimens and the loss of the caveolin-1 expression correlated with tumor progression, poor prognosis and drug resistance [14-16]. In our study, we observed equal amounts of caveolin-1 in tumor and normal tissues in 25% of samples, while in the others it was down-regulated. Although we did not find correlation of caveolin-1α expression with prognosis, we made an interesting observation. Seven out of 11 NSCLC samples with equal caveolin-1α protein expression in tumor and normal tissues and tumor size T1-T2 had lymph node metastases. This fact may be explained by the hypothesis of Ravid *et al.*[19], according to which the caveolin-1 expression is bi-phasic: i.e., it decreases at the early stages of the tumor transformation and increases later, at the stage of metastasis. According to the data published by Wiechen and Bayer-Garner, the majority of malignant STS are characterized by decreased amount of caveolin-1 protein [17,18]. Of special interest, in our opinion, are the results indicating that benign and malignant mesenchymal tumors differ by caveolin-1 expression. While in malignant neoplasms the expression of caveolin-1 is decreased, in benign tissue it is either increased or ‘normal’ levels of the protein are registered; this observation is also confirmed by the results of our study. We observed decreased protein expression of caveolin-1α in the majority of the malignant tumor specimens. At the same time, no decrease in expression of caveolin-1α has been demonstrated in 5 studied benign tumors.

### Correlations between mRNA expression levels of different MFP

Microdomain-forming proteins, due to the formation of signal platforms within the plasma membrane, are able to regulate a whole complex of intercellular pathways, and those represent an attractive target for chemotherapy. However, the relationships and mechanisms of possible interactions between different MFPs are poorly studied, although, they participate in common signal pathways [20]. The fact that animals with caveolin gene knock down are fertile and viable, whereas caveolin-1 is a key regulator of a wide range of vitally important pathways in the cell [21], may point to the existence of compensatory mechanisms or microdomain-forming backup proteins. This makes our study especially significant, as we were able to estimate changes of four MFPs simultaneously.

Statistical analysis using Spearman’s rank correlation test enabled us to reveal different correlations in NSCLC which are more typical for groups of tumors with favorable clinicopathological characteristics. We suggest a hypothesis according to which simultaneous changes in the MFPs mRNA expression characterize a presence of a certain *in vivo* regulatory relationships between proteins at early stages of tumor development. Progression of the disease (manifested in the increase of the size, decrease of the differentiation degree, ability to form secondary growth nodes) leads to an increasing misbalance of intercellular signaling pathways and loss of correlations between MFPs. On the other hand, the appearance of such strong correlations may be a consequence of a transcription regulation of the studied genes by common transcription factors. It is known that the transcription of caveolin-1 and flotillin-1 may be regulated by Sp1 and Ets-1 transcription factors [22-24]. We found that the strongest correlation in both studied groups of tumors was between mRNA expression of stomatin and flotillin-1, although, the expression patterns were diverse. This may indicate the existence of common mechanisms for their regulation in cells of epithelial and mesenchymal origin.

## Conclusion

In this study, we demonstrated that the expression of such MFPs as stomatin and flotillins changes in NSCLC and STS. Some of these changes correlate with clinicopathological characteristics, such as tumor size, differentiation degree, regional lymph node metastasis, and, correspondingly, the stage of the disease. Therefore, caveolin-1, stomatin and flotillins play an important role in the progression of both types of tumors. The discovery of correlations between mRNA expression of MFPs contributes to the understanding of regulation of these genes and may lead to a revision of the already accumulated scientific data. Our findings, which have demonstrated for the first time the role of stomatin in carcinogenic processes, open new avenues for future research on the functions of this protein, not only in the hematopoietic cells, but, primarily, in other types of cells, both in normal physiology and in pathology.

## Methods

### Ethics statement

The Institutional Review Board of N.N. Blokhin Russian Cancer Research Center of the Russian Academy of Medical Sciences approved the project and all patients, who were involved in the study, gave written informed consents that their samples could be used for investigational purposes. Data were analyzed anonymously. All potential participants who declined to participate or otherwise did not participate were eligible for treatment (if applicable) and were not disadvantaged in any other way by not participating in the study.

### Patients and specimens

Tumor tissue samples were obtained from 50 patients with NSCLC and 40 patients with STS, who had undergone surgery at the Clinical Oncology Research Institute, N.N. Blokhin RCRC RAMS between 2005 and 2007. The corresponding adjacent normal tissue samples (normal lung tissue for NSCLC and related mesenchymal tissue for STS) were also obtained. The tumor clinicopathological stages were determined according to the standard tumor TNM classification systems of the International Union Against Cancer (edition 6). The NSCLC group included 22 (44%) adenocarcinomas and 28 (56%) squamous cell carcinomas. There were 40 (80%) men and 10 (20%) women, with a median age of 60.82 years (range 38 – 79 years). Other specimens’ characteristics are presented in Table 6. The mesenchymal tumor group consisted of 15 liposarcomas (10 well-differentiated and 5 dedifferentiated), 7 malignant fibrous histiocytomas (six grade 3 and one grade 2), 6 synovial sarcomas (four grade 3 and two grade 2), 4 malignant schwannomas (two grade 3, one grade 1 and one grade 2), one leiomyosarcoma, one dermatofibrosarcoma, one spindle cell sarcoma and 5 benign tumors (three lipomas and two schwannomas). Sixteen tumors were located in soft tissues of the extremities, 21 in the retroperitoneal space, and 3 in the soft tissue of the trunk. There were 16 (40%) men and 24 (60%) women, with a median age of 52.33 years (range 17 – 81 years).

**Table 6:** Tumor characteristics in 50 cases of NSCLC

**Characteristics**		**No. (%) of cases**
Tumor stage	pT1	9 (18)
	pT2	23 (46)
	pT3	9 (18)
	pT4	9 (18)
Lymph node status	pN0	21 (42)
	pN1	13 (26)
	pN2	16 (32)
Distant metastasis	Absent	47 (94)
	Present	3 (6)
Clinical stage	I	14 (28)
	II	13 (26)
	III	20 (40)
	IV	3 (6)
Degree of differentiation	High	2 (4)
	Moderate	31 (62)
	Low	17 (34)

### Total RNA extraction and reverse transcriptase PCR

Frozen primary tumor specimens were homogenized in TRIzol reagent (Invitrogen) using a disrupter. Total RNA was extracted according to the TRIzol protocol. The total RNA of each sample was dissolved in RNase-free water and stored at −80°C. Before cDNA synthesis all the RNA samples were treated with DNase I (Fermentas) in order to avoid genomic DNA contamination. The RNA (2 μg) was reverse-transcribed in a 50 μl reaction using oligo-dT primers and MMLV-reverse transcriptase (Promega).

### Quantitative real-time PCR

Quantitative real-time PCR was performed on an iCycler iQ5 (Bio-Rad) using the EvaGreen dye. PCR reactions were carried out in a total volume of 25 μl containing 21.4 μl of PCR master mix, 3 μl of undiluted first-strand cDNA and 3 pmol of forward and reverse primers each. Sequences of the primers were as follows: caveolin-1, 5'-CCGCGACCCTAAACACCTC-3' (forward) and 5'-GCCTTCCAAATGCCGTCAA-3' (reverse); stomatin, 5'-GGGAGGGACGCATAGAAGGA-3' (forward) and 5'-GTACATTGTTGGAAAGGGAGGC-3' (reverse); flotillin-1, 5'-CTCCACCCCACCTCAACTTATTTA-3' (forward) and 5'-TCCAGCCCATCCCTCAGTCT-3' (reverse); GAPDH, 5'-TTGCCATGGGTGGAATCATA-3' (forward) and 5'-TCGGAGTCAACGGATTTGGT-3' (reverse). The following run protocol was used: denaturation step (95°C, 10 min), amplification and quantification programs repeated 45 times (95°C for 30 s, 60°C for 30 s and 72°C for 30 s). All the samples were amplified simultaneously in triplicate in a one assay-run. The transcript levels were normalized to those of GAPDH to account for variability in the amount of cDNA in each sample, and the relative expression levels were calculated using the REST-2005 software (Corbett Research/Qiagen) [25]. Genes with relative expression values greater than 1.5 or less than 0.5 were considered to be up- or down-regulated, respectively, in tumor tissues. Raw data are available in Additional file 1.

### Western blot analysis

Frozen primary tumor specimens were transferred into lysis buffer (10 mM Tris–HCl, pH 7.8, 100 mM NaCl, 10 mM EDTA, 1% Triton X-100, 10% glycerol, 0.1% SDS, 0,5% deoxycholate, protease inhibitor cocktail (Roche) and Halt phosphatase inhibitor cocktail (Thermo)) and incubated for 16 hours at +4°C. The lysates were centrifuged. Equal amounts of protein (80 μg) were separated by SDS-PAGE (10-12% separating gel) and transferred onto polyvinylidene difluoride membranes (Millipore). Immunodetection was performed using the caveolin-1α-specific monoclonal antibody (C3437, Sigma), the stomatin-specific monoclonal antibody (sc-134554, Santa Cruz), the flotillin-1-specific and flotillin-2-specific monoclonal antibody (clone 18 and clone 29, respectively, BD Transduction Laboratories), followed by chemiluminescent detection. (Millipore). To ensure equal loading amounts the blots were reprobed using a polyclonal antibody to pan-actin (Cell Signaling). The protein levels were quantified by densitometry using ImageJ software (NIH, Bethesda, MD, USA). These assessments were performed three times and after that tumor-to-normal protein abundance ratios were calculated. Protein expression was considered to be increased or decreased in tumor specimens if the ratio was less than 0.5 or more than 1.5, respectively. Raw data are available in Additional file 1.

### Statistical analysis

Statistical analysis was performed using IBM SPSS Statistics 19 software. The relationship between qualitative variables was analyzed using the χ2 or Fisher's exact test. Correlations between parameters were assessed according to the Spearman nonparametric test. Survival curves were plotted by the Kaplan-Meier method and compared using the log-rank test. The survival data were evaluated using univariate Cox regression analysis. The P-value of <0.05 was considered statistically significant.

## Competing interests

The authors declare that they have no competing interests.

## Authors’ contributions

KAA carried out molecular study and performed the statistical analysis, drafted the manuscript. ANS carried out molecular study and participated in the manuscript drafting. KKL provided information about the clinical specimens and participated in the statistical analysis. VVM collected the clinical samples. IBZ participated in the design of the study, coordination and the manuscript drafting. All authors read and approved the final manuscript.

## Pre-publication history

The pre-publication history for this paper can be accessed here:

http://www.biomedcentral.com/1471-2407/14/100/prepub

## Supplementary Material

Additional file 1: Table S1 Clinicopathological characteristics and microdomain-forming proteins expression in patients with non-small cell lung cancer. Table S2. Clinicopathological characteristics and microdomain-forming proteins expression in patients with soft tissue sarcomas. Click here for file
